# Machine learning-based model for predicting major adverse cardiovascular and cerebrovascular events in patients aged 65 years and older undergoing noncardiac surgery

**DOI:** 10.1186/s12877-023-04509-6

**Published:** 2023-12-07

**Authors:** Xuejiao Wu, Jiachen Hu, Jianjun Zhang

**Affiliations:** 1 Heart Center, Beijing Chaoyang Hospital, Capital Medical University, Beijing, 10020 China; 2 Department of Gastroenterology, Peking University Third Hospital, Beijing, 100191 China

**Keywords:** Cardiovascular events, Cerebrovascular events, Elderly patients, Prediction model, Risk assessment

## Abstract

**Background:**

Few evidence-based prediction models have been developed for predicting major adverse cardiovascular and cerebrovascular events (MACCE) in patients aged 65 years or older undergoing noncardiac surgery. In this study, we aimed to analyze the risk factors for perioperative MACCE in patients aged 65 years or older undergoing noncardiac surgery and construct a prediction model.

**Methods:**

In this nested case–control study, a total of 342 Chinese patients who were aged ≥ 65 years and underwent medium- or high-risk noncardiac surgery in our hospital were included. There were 84 cases with MACCE (the MACCE group) and 258 without MACCE (the control group). Univariable logistic regression analysis was performed to identify the risk factors for MACCE. Least absolute shrinkage and selection operator (LASSO) regression was used to screen the variables. Nomogram was constructed using the selected variables. Machine learning methods, including Decision Tree, XGBoost, Support Vector Machine, K-nearest Neighbor, and Neural network, was used to establish, validate, and compare the performance of different prediction models.

**Results:**

A prediction model based on nine variables, including age ≥ 85 years, history of ischemic chest pain, symptoms of decompensated heart failure, high-risk surgery, intraoperative minimum systolic blood pressure, postoperative systolic blood pressure, Cr levels over 2.0 mg/dL, left ventricular ejection fraction, and perioperative blood transfusion, was constructed. This LASSO logistic regression model showed good discriminatory ability to predict MACCE (area under the curve = 0.89; 95% confidence interval, 0.818 – 0.963) and fit to the test set (Hosmer–Lemeshow, χ2 = 7.4053, *P* = 0.4936). The decision curve analysis showed a positive net benefit of the new model. Compared with logistic regression model, the XGBoost model showed better prediction ability (area under the curve = 0.903). A preoperative prediction model based on five variables, including age ≥ 85 years, symptoms of decompensated heart failure, ischemic chest pain, high-risk type of surgery and Cr levels over 2.0 mg/dL was also constructed. This model showed good discriminatory ability to predict MACCE before surgery (area under the curve = 0.720 [95% CI, 0.591–0.848]. Both models compared with the modified RCRI score had improvement in reclassification.

**Conclusion:**

By analyzing Chinese patients aged ≥ 65 years undergoing medium- or high-risk noncardiac surgery, the risk factors for perioperative MACCE were identified. Then, simple prediction models were constructed and validated, which showed good prediction performance and may be used as a decision-making assistant tool for clinicians. These findings provide a basis for preventing and improving the perioperative management of MACCE.

## Background

With the increase in the aging population and the advancement in medical treatment, more elderly patients have received noncardiac surgery. However, previous studies and some guidelines have suggested that advanced age is an independent risk factor for major adverse cardiovascular [1–3] and cerebrovascular events [1, 4, 5] (MACCE) during the perioperative period of noncardiac surgery. The prevalence of comorbid conditions and the surgical types differ significantly between young and elderly patients [6]. The validated preoperative risk scoring system can be used to predict the risk of adverse events in patients undergoing noncardiac surgery. At present, three scoring systems for evaluating preoperative cardiac risk have been recognized in the guidelines: the modified Revised Cardiac Risk Index (RCRI), [7] the National Surgical Quality Improvement Program (NSQIP) Myocardial Infarction & Cardiac Arrest (MICA) calculator, [8] and the American College of Surgeons NSQIP (ACS NSQIP) Surgical Risk Calculator [9]. First of all, no currently available cardiac risk evaluation tools are designed for elderly patients. Secondly, the incidence rate of cerebrovascular events after non cardiac surgery is increasing significantly, but none of the three cardiac risk evaluation tools include cerebrovascular diseases as endpoints [10]. Thirdly,with the improvement in surgical methods and medical care in recent years, cardiac risk evaluation tools that were developed a long time ago need to be updated to meet the current clinical needs. -Finally, most cardiac risk evaluation tools have not been validated in the Chinese population. Therefore, it is of great significance to establish a model to predict the risk of MACCE during the perioperative period of noncardiac surgery for elderly Chinese patients.

In this study, we verified the effectiveness of the modified RCRI score recommended by the guidelines in the elderly population of China. Then we identified the risk factors for perioperative MACCE in elderly patients undergoing noncardiac surgery. We further established and validated new prediction models,and different prediction models were constructed and compared by machine learning methods. Lastly, we compared the new prediction models with modified RCRI score.

## Methods

### Ethical statement

This study was approved by the Ethics Committee of the Beijing Chaoyang Hospital (approval no. 2021-S-476). Informed consent was waived because of the retrospective nature of the study. All data were anonymous.

### Population and outcomes

All elective and elevated-risk surgeries requiring anesthesia that were performed at the Beijing Chaoyang Hospital between January 1st, 2018, and June 30th, 2022, were screened. According to the 2014 ESC/ESA Guidelines on non-cardiac surgery, elevated-risk surgery includes intermediate surgery (i.e., intraperitoneal surgery, carotid endarterectomy, peripheral arterioplasty, endovascular aneurysm repair, head and neck surgery, neurosurgery/major orthopedic surgery, neurosurgery/major orthopedic surgery, and major urological surgery) and high-risk surgery (i.e., aortic and other major vascular surgery, and peripheral vascular surgery) [3]. If the patient underwent more than one surgery in 4 months, only the first one was included. Patients were excluded if they (1) underwent transplantation or had traumatic injury; (2) were aged less than 65 years; (3) were at the American Society of Anesthesiologists Classification V; (4) underwent palliative surgery of advanced malignant tumor; (5) had congenital heart disease; (6) underwent cardiomyopathy or low-risk surgery (i.e., breast surgery, dental surgery, endoscopic procedure, ophthalmic surgery, gynecological surgery, reconstructive surgery, minor orthopedic surgery, and minor urological surgery). The main outcomes were the in-hospital risk of MACCE: acute myocardial infarction (AMI) [ICD-10 code I21], heart failure (HF) [ICD-10 codes I50], ischemic stroke [ICD-10 code I63 or I64], and all-cause death.

### Statistical analysis

Continuous variables were expressed as mean ± standard deviation and compared by the Mann–Whitney U test. Categorical variables were presented as absolute values and percentages and compared by the Chi-square test. There were missing values for predictors in this study, which were handled by multiple imputation with five imputations, and all predictor variables were included in the imputation model. After imputation was completed, the results of the analysis of the imputed data were merged according to Rubin's rules. Univariable logistic regression analyses were applied to identify the risk factors for MACCE during the perioperative period of high-risk noncardiac surgery in elderly patients. LASSO regression was used to screen the variables. Nomograms were constructed using the selected variables. The receiver operating characteristic (ROC) curve was plotted to evaluate the discriminatory ability of the nomograph model. The Hosmer–Lemeshow test was used to evaluate the goodness-of-fit of the models and the calibration curves were plotted. The net benefit was estimated by the decision curve analysis. Using the Areas under the receiver operating characteristic curves (AUC), the unclassified net reclassification improvement (NRI) and integrated discrimination improvement (IDI), we compared the performance of our model to the modified RCRI score. We employed five machine learning methods—Decision Tree, XGBoost, Support Vector Machine, K-nearest Neighbor, and Neural network(R package:rpart,rpart.plot,xgboost,e1071,sklearn,neuralnet)—for model establishment. A sample of 70% of the cohort generated randomly using a seed was applied for the training set; the remaining 30% was used for testing. The R software 4.0.2 was used for data analysis. *P* < 0.05 indicated statistical significance.

## Results

### MACCE and mortality

From January 1st, 2018, to June 30th, 2022, 40873 adult patients underwent surgery in our hospital. After screening, a total of 9739 patients were included. Among them, 84 cases had MACCE events. The incidence of MACCE and the mortality rate of patients undergoing noncardiac surgery increased with age (Table 1). In patients aged ≥ 85 years, the incidence of MACCE was 2.462% and the mortality rate was 0.821%.

**Table 1:** The incidence of MACCE and the mortality rate of patients at different ages

Age	Number of total population	Numbers of AMI (% of total population)	Numbers of HF (% of total population)	Numbers of stroke (% of total population)	Numbers of death (% of total population)	Numbers of MACCE (% of total population)
65–74 years	5315	4 (0.075%)	7 (0.132%)	7 (0.132%)	9 (0.169%)	25 (0.470%)
75–84 years	3449	5 (0.145%)	28 (0.812%)	2 (0.058%)	3 (0.087%)	35 (1.015%)
≥ 85 years	975	7 (0.718%)	13 (1.333%)	3 (0.307%)	8 (0.821%)	24 (2.462%)

*Abbreviations*: *AMI* acute myocardial infarction, *HF* heart failure, *MACCE* major adverse cardiovascular and cerebrovascular even

Patients without MACCE during hospitalization were firstly divided into five subgroups according to the operation type of the MACCE group: intraperitoneal surgery, thoracic surgery, peripheral arterioplasty, major orthopedic surgery and major urological surgery. Stratified randomization was used to protect against the possibility of imbalance with the MACCE group. Then, 285 cases stratified by the five subgroups were randomly selected as the control group. Finally, 342 patients (84 in the MACCE group and 258 in the control group) were included for further analysis.

3.2 Patient characteristics and verification of the modified RCRI scoreThe baseline characteristics of the MACCE and control groups are shown in Table 2. The mean age was significantly different between the two groups. The proportion of patients aged ≥ 85 years was significantly higher in the MACCE group than in the control group (*P* = 0.003). Compared to the controls, significantly more patients in the MACCE group had a history of coronary heart disease, ischemic chest pain, HF (especially HF with symptoms), arrhythmia, atrial fibrillation, valvular heart disease, nitrate therapy, and creatinine (Cr) levels of over 2.0 mg/dL (all *P* > 0.05). The blood transfusion and blood transfusion volume, intraoperative minimum diastolic pressure, minimum mean arterial pressure, intraoperative maximum heart rate, postoperative systolic pressure, and postoperative heart rate of the MACCE group were significantly different from those of the control group, while no significant difference was observed in the high-risk surgery type. Compared with the control group, patients with MACCE showed significantly larger left atrial anterior–posterior diameter, left atrial transverse diameter, left atrial long diameter, left ventricular mass, and left ventricular mass index, and lower left ventricular ejection fraction (LVEF).

**Table 2:** Baseline characteristics of recruited patients

Characteristics	All (*n* = 342)	Control group (*n* = 258)	MACCE group (*n* = 84)	*P*
Age (mean (SD))	77.05 (7.53)	76.54 (7.50)	78.63 (7.46)	0.027
Age 65–74 years (%)	129 (37.7)	104 (40.3)	25 (29.8)	0.109
Age 75–84 years (%)	147 (43.0)	112 (43.4)	35 (41.7)	0.878
Age ≥ 85 years (%)	59 (17.3)	35 (13.6)	24 (28.6)	0.003
Height (mean (SD))	162.85 (8.65)	163.03 (8.60)	162.31 (8.84)	0.51
Weight (mean (SD))	63.59 (12.20)	63.97 (11.99)	62.40 (12.84)	0.307
Chronic respiratory disease (%)	33 (9.6)	23 (8.9)	10 (11.9)	0.553
CAD (%)	96 (28.1)	63 (24.4)	33 (39.3)	0.013
Positive exercise test (%)	12 (3.5)	7 (2.7)	5 (6.0)	0.289
Ischemic chest pain (%)	16 (4.7)	3 (1.2)	13 (15.5)	< 0.001
CHF (%)	19 (5.6)	4 (1.6)	15 (17.9)	< 0.001
Symptoms of decompensated HF (%)	15 (4.4)	2 (0.8)	13 (15.5)	< 0.001
Arrhythmia (%)	48 (14.0)	26 (10.1)	22 (26.2)	< 0.001
AF (%)	43 (12.6)	22 (8.5)	21 (25.0)	< 0.001
Nitrate therapy (%)	33 (9.6)	17 (6.6)	16 (19.0)	0.002
VHD (%)	25 (7.3)	8 (3.1)	17 (20.2)	< 0.001
Cr > 2.0 mg/dL (mean (SD))	0.04 (0.20)	0.02 (0.15)	0.10 (0.30)	0.004
Blood transfusion (%)	166 (48.5)	115 (44.6)	51 (60.7)	0.014
The amount of transfused blood (mean (SD)	673.13 (1650.54)	428.52 (650.81)	1424.40 (3020.58)	< 0.001
High-risk type of surgery (mean (SD))	0.37 (0.48)	0.34 (0.47)	0.45 (0.50)	0.067
Intraperitoneal surgery (%)	95 (27.8)	66 (25.6)	29 (34.5)	0.147
General anesthesia (%)	193 (56.4)	138 (53.5)	55 (65.5)	0.072
NNIS class				
0 (%)	115 (33.6)	92 (35.7)	23 (27.4)	0.207
1 (%)	151 (44.2)	108 (41.9)	43 (51.2)	0.171
2 (%)	21 (6.1)	10 (3.9)	11 (13.1)	0.005
3 (%)	4 (1.2)	2 (0.8)	2 (2.4)	0.545
Preoperative heart rate (mean (SD))	72.45 (47.88)	69.08 (13.92)	82.79 (93.14)	0.022
Postoperative systolic blood pressure (mean (SD))	138.89 (22.38)	141.15 (21.72)	131.98 (23.07)	0.001
Postoperative heart rate (mean (SD))	79.05 (16.05)	77.69 (13.10)	83.21 (22.43)	0.006
Intraoperative minimum systolic blood pressure (mean (SD))	112.04 (21.28)	113.45 (20.59)	107.70 (22.85)	0.031
Intraoperative minimum diastolic pressure (mean (SD))	54.25 (12.78)	55.22 (11.91)	51.25 (14.82)	0.013
Minimum mean arterial pressure (mean (SD))	73.10 (14.41)	74.25 (13.85)	69.58 (15.58)	0.01
Intraoperative maximum heart rate (mean (SD))	84.60 (18.97)	82.44 (17.01)	91.23 (22.89)	< 0.001
Sinus rhythm (%)	312 (91.2)	241 (93.4)	71 (84.5)	0.023
ECG with Q waves (%)	43 (12.6)	31 (12.0)	12 (14.3)	0.722
Myocardial ischemia changes in ECG (%)	87 (25.4)	58 (22.5)	29 (34.5)	0.04
L (mean (SD))	1.43 (0.68)	1.48 (0.70)	1.30 (0.61)	0.046
ALT (mean (SD))	26.65 (42.76)	23.23 (30.70)	37.17 (66.66)	0.009
ApoA-I (mean (SD))	274.33 (269.20)	258.55 (260.51)	322.81 (290.57)	0.057
Left atrium diameter (mean (SD))	35.54 (5.17)	35.05 (4.90)	37.05 (5.67)	0.002
Left ventricle transverse diameter (mean (SD))	35.65 (5.74)	35.05 (5.24)	37.48 (6.79)	0.001
Left ventricular dilatation (mean (SD)	0.12 (0.33)	0.11 (0.31)	0.17 (0.37)	0.159
LVEF (mean (SD))	66.94 (7.05)	67.82 (5.86)	64.25 (9.40)	< 0.001
Left ventricle long diameter (mean (SD))	51.18 (7.41)	50.33 (6.73)	53.80 (8.75)	< 0.001
LVDd (mean (SD))	46.82 (4.49)	46.64 (3.94)	47.39 (5.85)	0.18
Posterior wall thickness of left ventric (mean (SD))	9.79 (1.15)	9.78 (1.14)	9.81 (1.16)	0.793
IVST (mean (SD))	10.37 (1.70)	10.27 (1.19)	10.68 (2.71)	0.053
LVM I (mean (SD)	105.70 (31.05)	103.53 (27.29)	112.34 (39.95)	0.024
Left ventricular mass (LVM) (mean (SD)	168.21 (43.65)	165.41 (37.56)	176.82 (57.96)	0.037
MACCE		0	84 (100%)	< 0.001
AMI		0	16 (19.05%)	
HF		0	48 (57.14%)	
Stroke		0	12 (14.29%)	
Death		0	20 (23.81%)	

*Abbreviations*: *CAD* coronary heart disease, *CHF* Chronic heart failure, *AF* atrial fibrillation, *VHD* Valvular heart disease, *NNIS class* National Nosocomial Infections Surveillance, *ECG* electrocardiogram, *Cr* creatinine, *L* Lymphocyte count, *ALT* glutamic-pyruvic transaminase, *LVEF* Left ventricular ejection fraction, *LVDd* Left ventricular end-diastolic internal diameter, *IVST* Left ventricular mass index, *LVMI* Left ventricular mass index, *MACCE* major adverse cardiovascular and cerebrovascular even, *AMI* acute myocardial infarction, *HF* heart failure

Since the modified RCRI score did not take ischemic stroke and non-cardiac death into consideration, we validated the modified RCRI score after excluding 26 patients who experienced ischemic stroke or died due to non-cardiac cause during hospitalization. The area under the curve (AUC) of the modified RCRI score predicting the occurrence of MACCE in elderly patients undergoing noncardiac surgery in our study was 0.54 [95% confidence interval (0.419, 0.660)].

### Risk factors

Univariable logistic regression analysis showed that the risk factors for perioperative MACCE in patients aged ≥ 65 years (Table 3) were as follows: age (especially ≥ 85 years), medical history (i.e., history of coronary heart disease, ischemic chest pain, nitrate therapy, HF, arrhythmia, heart valve disease, and Cr levels of over 2.0 mg/dL), blood transfusion, the amount of transfused blood, surgery conditions (i.e., high-risk surgery type, intraoperative minimum systolic blood pressure, intraoperative minimum diastolic blood pressure, intraoperative minimum mean arterial pressure, intraoperative maximum heart rate, postoperative systolic blood pressure, and postoperative heart rate), laboratory test results (i.e., ApoA-I, myocardial ischemia changes in electrocardiogram), left atrial anterior–posterior diameter, left atrial transverse diameter, left atrial long diameter, and LVEF).

**Table 3:** Univariablelogistic analysis of risk factors for perioperative MACCE

Characteristics	OR	CI.2.5.	CI.97.5.	*P*
Age	1.038	0.998	1.081	0.0682
Age 65–74 years	0.594	0.312	1.096	0.1026
Age 75–84 years	0.984	0.544	1.766	0.9572
Age ≥ 85 years	2.495	1.22	5.041	0.0111
Blood transfusion	1.561	0.872	2.814	0.135
General anesthesia	1.533	0.848	2.824	0.1627
NNIS Class				
0	0.657	0.348	1.206	0.1829
1	1.678	0.936	3.025	0.0827
2	4.117	0.883	21.42	0.0692
3	6.034	0.568	131.225	0.1452
Sinus rhythm	0.49	0.203	1.238	0.1179
Insulin	0.591	0.166	1.652	0.3572
Stroke/TIA	1.276	0.64	2.469	0.4766
Chronic respiratory disease	1.321	0.486	3.273	0.562
CAD	1.857	0.992	3.442	0.0502
Positive exercise test	2.442	0.587	9.532	0.1943
Ischemic chest pain	19.47	5.022	128.512	0.0002
CHF	14.367	4.362	64.898	0.0001
Symptoms of decompensated HF	26.868	4.777	504.118	0.0021
Arrhythmia	3.254	1.552	6.804	0.0016
AF	3.322	1.518	7.26	0.0024
Reduced LVEF	15.893	2.495	307.917	0.0124
VHD	6.824	2.316	22.752	0.0008
ECG with Q waves	1.947	0.869	4.221	0.0958
Myocardial ischemia changes in ECG	1.824	0.958	3.427	0.0633
Nitrate therapy	4.139	1.688	10.369	0.0019
Intraperitoneal surgery	1.66	0.876	3.102	0.1145
Cr > 2.0 mg/dL	6.4	1.633	31.119	0.0103
The amount of transfused blood	1	1	1.001	0.0139
Preoperative heart rate	1.013	1	1.032	0.1921
Postoperative systolic blood pressure	0.987	0.974	1	0.058
Postoperative pluse	1.01	0.991	1.029	0.3109
Intraoperative minimum systolic blood pressure	0.99	0.974	1.005	0.1801
Intraoperative minimum diastolic pressure	0.976	0.951	0.999	0.052
Minimum mean arterial pressure	0.98	0.958	1.001	0.0677
Intraoperative maximum heart rate	1.018	1.003	1.034	0.0195
L	0.693	0.43	1.074	0.1151
ALT	1.004	0.998	1.011	0.2251
ApoA-I	1.001	1	1.002	0.0304
Left atrium diameter	1.069	1.011	1.132	0.0187
Left—ventricle transverse diameter	1.064	1.013	1.118	0.013
Left ventricular dilatation	1.457	0.596	3.342	0.3865
LVEF	0.929	0.887	0.969	0.001
High-risk type of surgery	1.891	1.044	3.424	0.035
Left—ventricle long diameter	1.068	1.025	1.115	0.0021
LVDd	1.029	0.965	1.098	0.381
Posterior wall thickness of left ventric	0.995	0.776	1.276	0.971
IVST	1.093	0.893	1.337	0.3718
Height	1.005	0.972	1.04	0.7513
Weight	0.991	0.967	1.015	0.4555
LVMI	1.004	0.996	1.013	0.3111
Left ventricular mass	1.004	0.997	1.01	0.2773

*Abbreviations*: *NNIS class* National Nosocomial Infections Surveillance, *TIA* Transient ischemic attack, *CAD* coronary heart disease, *CHF* Chronic heart failure, *AF* atrial fibrillation, *LVEF* Left ventricular ejection fraction, *VHD* Valvular heart disease, *ECG* electrocardiogram, *Cr* creatinine, *L* Lymphocyte count, *ALT* glutamic-pyruvic transaminase, *LVDd* Left ventricular end-diastolic internal diameter, *IVST* Left ventricular mass index, *LVMI* Left ventricular mass index

### Contribution and performance of the new prediction nomogram

**Fig. 1:**
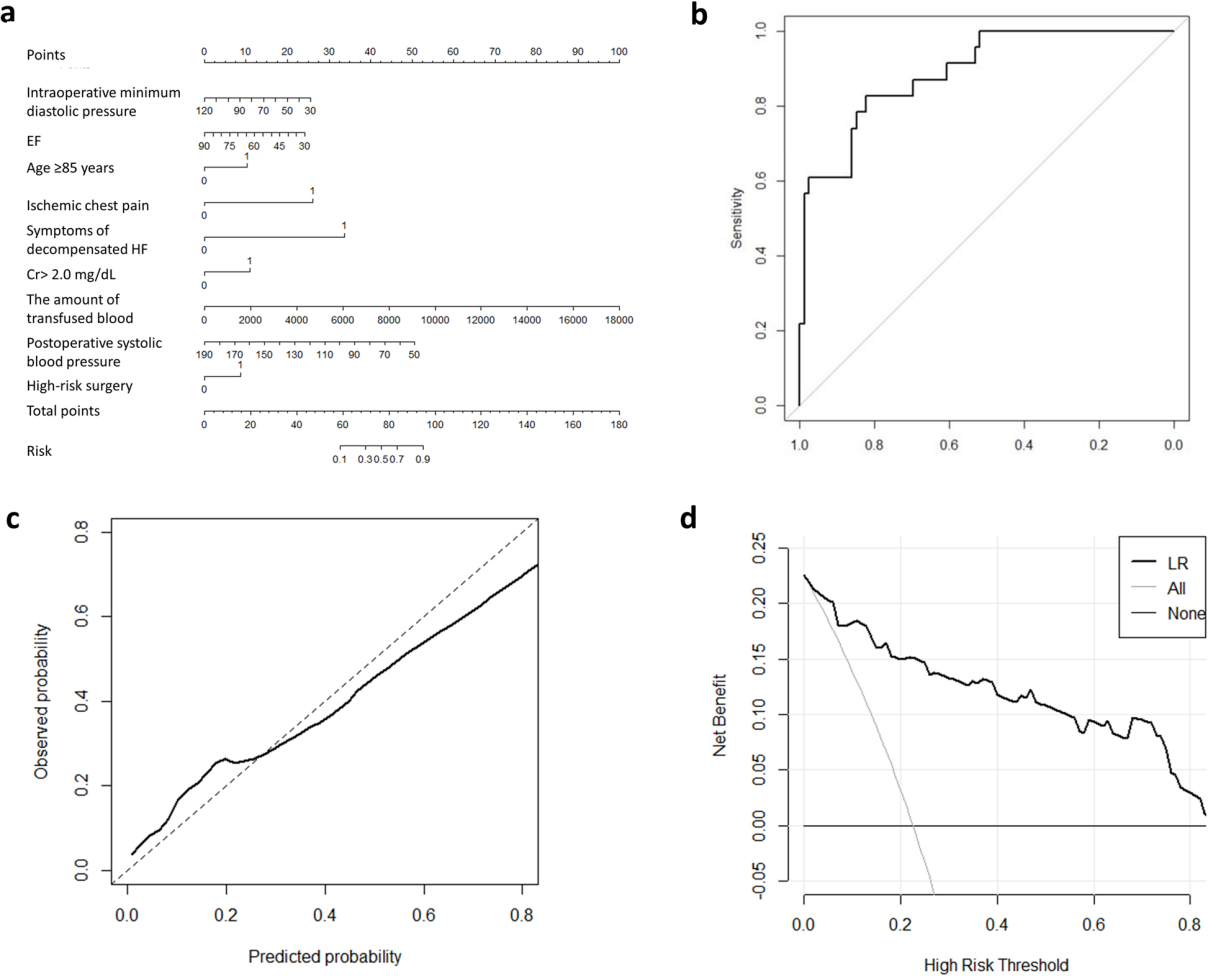
The nomogram, ROC curve, calibration plot, and the decision curve of the new predictive model. **a** Images indicating the nomogram for each variable of the prediction model; **b** ROC curve of the new predictive model with the validation cohort; **c** Calibration plot for the new prediction model with the validation cohort; **d** Decision curve of the new predictive model. EF: Left ventricular ejection fraction;Cr 2.0 > mg/dL, preoperative serum creatinine > 2.0 mg/dL

LASSO regression analysis revealed that age ≥ 85 years, history of ischemic chest pain, symptoms of decompensated heart failure, high-risk surgery, intraoperative minimum systolic blood pressure, postoperative systolic blood pressure, Cr levels over 2.0 mg/dL, left ventricular ejection fraction, and perioperative blood transfusion were risk factors for perioperative MACCE in elderly patients. An optimal nomograph model for predicting the risk of perioperative MACCE in elderly patients undergoing noncardiac surgery was constructed using the training set (70% of all recruited patients; 60 in the MACCE group and 181 in the control group) (Fig. 1a). The sum of the score of each variable was considered the total score of the patient, and a vertical line was drawn at the total score. The corresponding prediction probability was defined as the perioperative incidence of MACCE in patients with noncardiac surgery.

The nomogram was internally validated by the test set population (30% of all recruited patients). The area under the curve (AUC) of the nomogram model predicting the occurrence of MACCE in elderly patients undergoing noncardiac surgery was 0.89 [95% confidence interval (0.818, 0.963)] (Fig. 1b). The calibration curve of the nomogram model was consistent with the actual curve, and it was confirmed by the Hosmer–Lemeshow goodness of fit test (χ2 = 4.9299, *P* = 0.765, Fig. 1c). The decision curve showed a large net benefit across the range of the MACCE risk of the new prediction nomogram (Fig. 1d).Compared with the modified RCRI score, the new prediction model had significant improvement in reclassification as assessed by the NRI (1.08 [95% CI, 0.835–1.334]) and IDI (0.307 [95% CI, 0.231–0.383]).

**Fig. 2:**
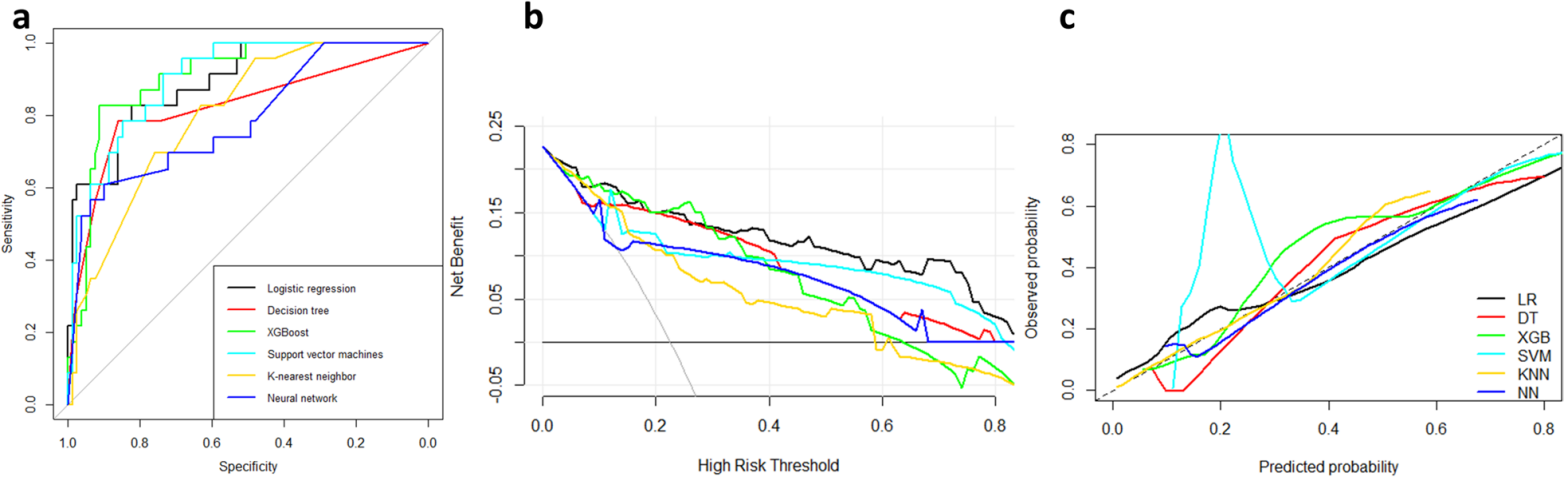
Comparison between the LASSO logistic regression and five machine learning methods. **a** AUC of the six methods. **b** Decision curve analysis evaluation of six methods. **c** Calibration plot of the six methods

Five machine learning methods, including Decision Tree, XGBoost, Support Vector Machine, K-nearest Neighbor, and Neural network were used to establish prediction models using the training set, which were then validated on the test set. The five methods and the LASSO logistic regression results were compared with the ROC curve, calibration plot, decision curve, AUC,sensitivity, specificity, and F1 score of the test set (Fig. 2, Table 4). The XGBoost model was the best model when the same variables were selected (AUC = 0.903, Accuracy = 0.892).

**Table 4:** Performance evaluation and comparison of six models

	Logistic regression	Decision Tree	XGBoost	Support Vector Machine	K-nearest Neighbor	Neural Network
AUC	0.891 [0.818–0.963]	0.824 [0.718–0.930]	0.903 [0.839–0.968]	0.901 [0.839–0.963]	0.803 [0.712–0.894]	0.789 [0.677–0.900]
Sensitivity	0.8261	0.7826	0.8261	0.9130	0.8261	0.6087
Specificity	0.8228	0.8608	0.9114	0.7342	0.6329	0.8987
Accuracy	0.824 [0.736–0.892]	0.843 [0.758–0.908]	0.892 [0.815–0.945]	0.775 [0.681–0.851]	0.677 [0.577–0.766]	0.833 [0.747–0.900]
Pos Pred Value	0.5758	0.6207	0.7308	0.5000	0.3958	0.6364
Neg Pred Value	0.9420	0.6207	0.9474	0.9667	0.9259	0.8875
F1 score	0.6786	0.6923	0.7755	0.6462	0.5352	0.622

**Fig. 3:**
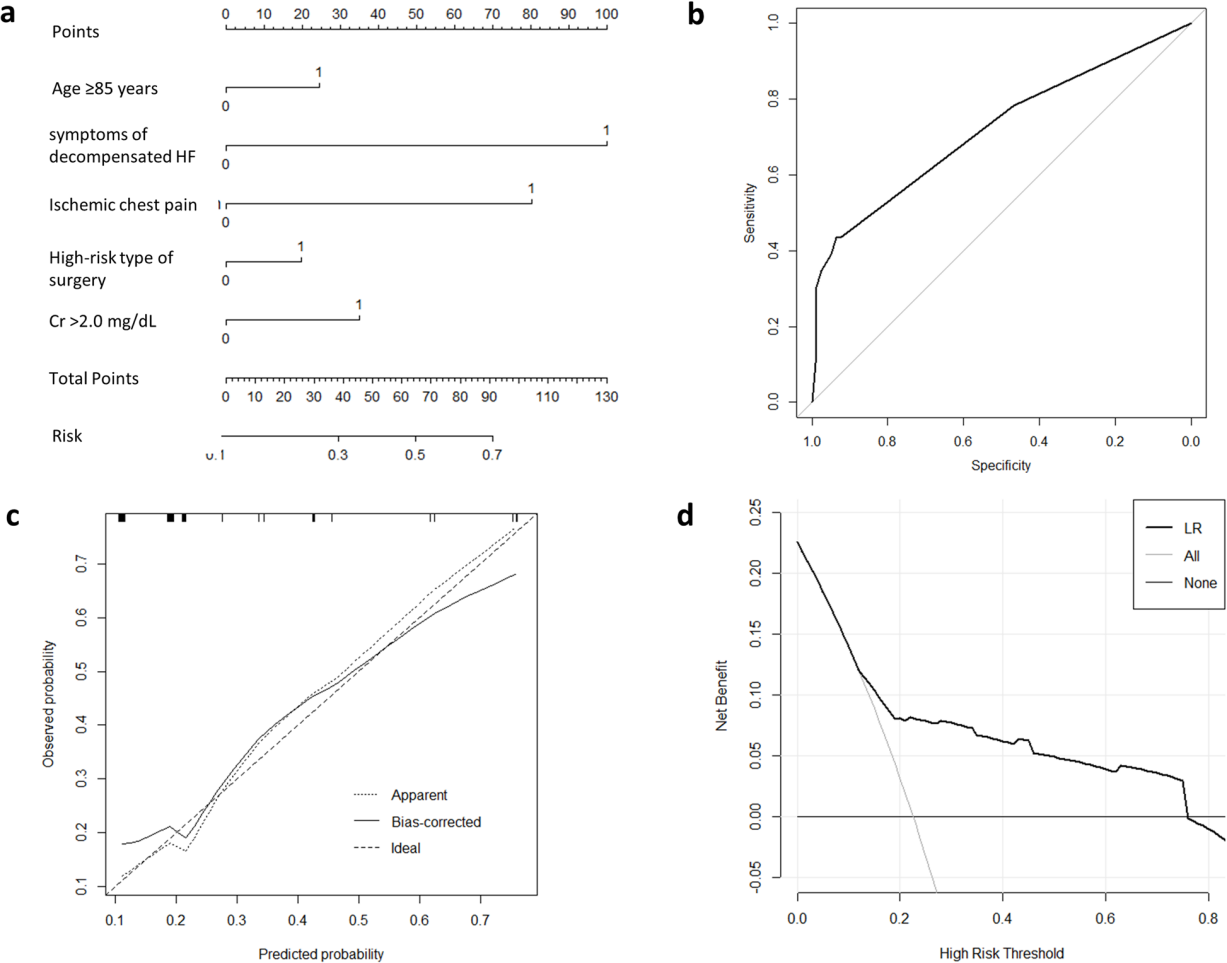
The nomogram, ROC curve, calibration plot, and the decision curve of the preoperative predictive model. **a** Images indicating the nomogram for each variable of the preoperative predictive model; **b** ROC curve of the preoperative predictive model with the validation cohort; **c** Calibration plot for the preoperative predictive model with the validation cohort; **d** Decision curve of the preoperative predictive model. Cr 2.0 > mg/dL, preoperative serum creatinine > 2.0 mg/dL

In order to guide pre-operative cardiac evaluation and optimization of patients at elevated risk prior to surgery, we reconstructed the preoperative prediction model after removing the intraoperative and postoperative variables. Using LASSO logistic regression, we selected 5 strongest predictors: age ≥ 85 years, symptoms of decompensated HF, ischemic chest pain, high-risk type of surgery and Cr > 2.0 mg/dL. Integrating the 5 variables, we were able to build a nomogram for predicting in-hospital MACCEs using the training set (70% of all recruited patients) (Fig. 3a). The preoperative prediction nomogram was internally validated by the test set population (30% of all recruited patients). The ROC curve of the preoperative prediction nomogram is shown in Fig. 3b with an AUC of 0.720 [95% CI, 0.591–0.848] and the accuracy is 0.824 [95% CI, 0.735–0.892]. The sensitivity of preoperative prediction nomogram is 0.435 and the specificity is 0.937. The calibration plot of the preoperative model showed an overall good agreement between the predicted and observed risks, which was further supported by Hosmer–Lemeshow test (Fig. 3c, χ2 = 0. 857, *p* = 0.999). The decision curve showed a large net benefit across the range of the MACCE risk of the preoperative prediction nomogram (Fig. 3d).Compared with the modified RCRI score, the preoperative prediction model had improvement in reclassification as assessed by the NRI (0.639 [95% CI, 0.369–0.909]) and IDI (0.235[95% CI, 0.162–0.308]). But compared with the preoperative prediction model, our nomogram with intraoperative and postoperative variables were better in reclassification as assessed by the NRI (0.609 [95% CI, 0.330–0.888]) and IDI (0.071[95% CI, 0.028–0.115]).

## Discussion

Elderly patients undergoing noncardiac surgery have a higher chance of developing complications, such as surgical complications, and having worse general conditions compared to young and middle-aged patients [11, 12]. In this cohort, the incidence of perioperative MACCE and the mortality rate increased significantly with age. In patients aged ≥ 85 years, the incidence of perioperative MACCE and the mortality rate were as high as 2.463% and 0.821%, respectively. Therefore, more attention needs to be paid to the risk of perioperative MACCE in elderly patients undergoing high-risk noncardiac surgery.

The risk factors identified in this study for MACCE in elderly patients undergoing medium and high-risk noncardiac surgery are in line with previous reports. It is worth noting that blood transfusion and the amount of blood transfusion are independent predictors of perioperative MACCE [13]. Most patients who need blood transfusion and a large amount of blood transfusion have more pre-existing diseases (e.g., anemia), a larger surgical wound (e.g., after Whipple surgery), and more intraoperative and postoperative blood loss, all of which may increase the incidence of MACCE. Secondly, blood pressure plays a key role in maintaining the perfusion of all organs. Therefore, blood pressure management during the perioperative period is important. Previous studies have shown that hypotension is closely related to myocardial injury, myocardial infarction, renal injury, and death [14, 15]. The Perioperative Quality Initiative consensus statement on intraoperative blood pressure, risks, and outcomes of elective noncardiac surgery suggests that hypotension, regardless of its cause, even brief hypotension, is harmful [16]. In this study, we found that intraoperative minimum diastolic pressure, intraoperative minimum systolic pressure, intraoperative minimum mean arterial pressure, and postoperative systolic pressure of the MACCE group were significantly different from those of the controls. Thirdly, univariable logistic regression analysis demonstrated that high heart rate during surgery was a risk factor for MACCE, possibly due to increased myocardial oxygen consumption as the heart rate increases. These findings need to be validated in large-scale studies.

In this study, we further constructed and validated a model to predict the risk of perioperative MACCE in elderly patients undergoing medium and high-risk noncardiac surgery. The prediction model not only showed good prediction performance but also exhibited the following characteristics. Firstly, fewer predictive variables were included in this model, which improved its clinical applicability. The ACS NSQIP Surgical Risk Calculator contains 22 variables and requires a network calculator, while this prediction model only contains 9 variables and can be calculated by nomogram. Secondly, the included variables are easy to obtain in clinical settings, including age ≥ 85 years, medical history of ischemic chest pain and symptoms of decompensated HF, high-risk surgery, intraoperative minimum systolic blood pressure and postoperative systolic blood pressure, Cr levels over 2.0 mg/dL, LVEF, and perioperative blood transfusion. The levels of Cr can be measured in the routine renal function examination before surgery. LVEF can be detected by preoperative echocardiography without affecting patient’s condition, medical insurance cost, and the average length of stay. Thirdly, compared with RCRI and ACS NSQIP Surgical Risk Calculator, our model also included blood pressure and LVEF. LVEF is an independent influencing factor for perioperative adverse events and long-term mortality risk [17–19]. In contrast to medical history and clinical symptoms, LVEF is an objective variable. Lastly, the endpoints of this prediction model included ischemic stroke in addition to major adverse cardiovascular events. With the increase in the incidence rate of perioperative ischemic stroke in recent years, this model may better meet the clinical needs.

Machine learning is a subfield of artificial intelligence, which effectively learns the features of a large input data set and provides an alternative method for risk prediction. In this study, five algorithms based on machine learning, including Decision Tree, XGBoost, Support Vector Machine, K-nearest Neighbor, and Neural Network, were used to evaluate the prediction model for the perioperative risk of MACCE. The prediction ability of the XGBoost model was slightly better than that of the Lasso logistic regression model (AUC = 0.903) when the same variables were selected.

This study also established a preoperative prediction model to facilitate clinicians' preoperative risk assessment. Combining the two models, preoperative and postoperative, could lead to a better risk assessment and postoperative management. Both models compared with the modified RCRI score had improvement in reclassification.

This study has some limitations. First, it was a single-center study, and the results were not externally validated. Secondly, the sample size was small due to the low prevalence of MACCE. Even if the random sampling method was adopted, selection bias might occur. Further studies with a larger sample size are needed. Thirdly, although compared with the preoperative prediction model, the prediction model with intraoperative and postoperative variables was better in reclassification as assessed by the NRI and IDI, but the utility of it is limited. Finally, this was a retrospective study. Further studies with a prospective design are needed to explore the potential risk factors for perioperative MACCE and to establish a model with better prediction performance.

## Conclusion

This study identified the risk factors for perioperative MACCE in elderly patients undergoing medium and high-risk noncardiac surgery, which provided a basis for better management and prevention of MACCE. A new model for predicting the risk of perioperative MACCE was constructed and validated. It showed good prediction performance and may be used as a decision-making assistant tool for clinicians. These data lay a foundation for future large-scale prospective clinical trials.

## Data Availability

All data generated or analyzed during this study are included in this published article.

## Abbreviations

MACCE: Major adverse cardiovascular and cerebrovascular even
CAD: Coronary heart disease
CHF: Chronic heart failure
AF: Atrial fibrillation
VHD: Valvular heart disease
NNIS class: National Nosocomial Infections Surveillance
ECG: Electrocardiogram
Cr: Creatinine
L: Lymphocyte count
ALT: Glutamic-pyruvic transaminase
LVEF: Left ventricular ejection fraction
LVDd: Left ventricular end-diastolic internal diameter
IVST: Left ventricular mass index
LVMI: Left ventricular mass index
AMI: Acute myocardial infarction
HF: Heart failure
